# *rst* Transcriptional Activity Influences kirre mRNA Concentration in the Drosophila Pupal Retina during the Final Steps of Ommatidial Patterning

**DOI:** 10.1371/journal.pone.0022536

**Published:** 2011-08-08

**Authors:** Maiaro Cabral Rosa Machado, Shirlei Octacilio-Silva, Mara Silvia A. Costa, Ricardo Guelerman P. Ramos

**Affiliations:** Departmento de Biologia Celular e Molecular e Bioagentes Patogênicos, Faculdade de Medicina de Ribeirão Preto, Universidade de São Paulo, Ribeirão Preto, São Paulo, Brazil; University of Dayton, United States of America

## Abstract

**Background:**

Drosophila retinal architecture is laid down between 24–48 hours after puparium formation, when some of the still uncommitted interommatidial cells (IOCs) are recruited to become secondary and tertiary pigment cells while the remaining ones undergo apoptosis. This choice between survival and death requires the product of the *roughest* (*rst*) gene, an immunoglobulin superfamily transmembrane glycoprotein involved in a wide range of developmental processes. Both temporal misexpression of Rst and truncation of the protein intracytoplasmic domain, lead to severe defects in which IOCs either remain mostly undifferentiated and die late and erratically or, instead, differentiate into extra pigment cells. Intriguingly, mutants not expressing wild type protein often have normal or very mild rough eyes.

**Methodology/Principal Findings:**

By using quantitative real time PCR to examine *rst* transcriptional dynamics in the pupal retina, both in wild type and mutant alleles we showed that tightly regulated temporal changes in *rst* transcriptional rate underlie its proper function during the final steps of eye patterning. Furthermore we demonstrated that the unexpected wild type eye phenotype of mutant*s* with low or no *rst* expression correlates with an upregulation in the mRNA levels of the *rst* paralogue *kin-of-irre* (*kirre*), which seems able to substitute for *rst* function in this process, similarly to their role in myoblast fusion. This compensatory upregulation of kirre mRNA levels could be directly induced in wild type pupa upon RNAi-mediated silencing of *rst*, indicating that expression of both genes is also coordinately regulated in physiological conditions.

**Conclusions/Significance:**

These findings suggest a general mechanism by which *rst* and *kirre* expression could be fine tuned to optimize their redundant roles during development and provide a clearer picture of how the specification of survival and apoptotic fates by differential cell adhesion during the final steps of retinal morphogenesis in insects are controlled at the transcriptional level.

## Introduction

Differential adhesion plays a central role in morphogenesis, allowing cells to sort themselves out from initially unpatterned populations as they become committed to specific fates [1], [2], and helping a wide range of cell types, such as neurons, lymphocytes and neural crest cells to navigate and find their interacting partners with exquisite precision [3], [4]. Besides, it is central to the molecular mechanisms mediating changes in tissue organization and shape, both in physiological, and pathological contexts [5]–[7].

Adhesive properties of cells are critically dependent on a highly diverse group of cell surface adhesion molecules, or CAMs, that can specifically bind to similar or different CAMs present either in other cells or in the extracellular matrix. At any given time each cell expresses a characteristic subset of CAMs that define both the specificity and mechanical strength of these interactions. Additionally a significant number of CAM families are composed of transmembrane glycoproteins whose intracellular domains also interact with the cell cytoskeleton, thus being able to mediate complex changes in cell shape upon binding or dissociating from their ligands. Among these, an important, albeit comparatively little studied group is the *Irre Recognition Module* or IRM [8], a small set of evolutionarily conserved transmembrane glycoproteins belonging to the immunoglobulin superfamily. IRM proteins can be further divided in two classes according to their structural and interacting properties: One group with shorter extracellular domains containing five immunoglobulin-like (Igl) repeats, whose prototypical members are the products of the *roughest* (*rst*) and *kin-of-irre* (*kirre*) paralogue genes of Drosophila [9]–[11], the mammalian Nephs [12], [13] and *C. elegans* SYG-1 [14]; and another, having longer ectodomains with eight or nine Igl repeats and one fibronectin type III domain, of which the best studied members are Nephrin in humans [15], Stick-and-Stones (Sns) and Hibris (Hbs) in Drosophila [16]–[18] and *C. elegans* SYG-2 [19]. Recent work in several laboratories has shown that tightly regulated interactions between members of the two classes appear to underlie the involvement of IRM proteins in a wide range of developmental processes throughout the phylogenetic tree [19]–[24]. In Drosophila, these processes include axonal pathfinding [9], [25], [26] myoblast cell fusion [10], [11], [16]–[18] sensory organ spacing [8], [27], embryonic midline integrity [28; Moda LMR and Ramos RGP unpublished observations], salivary gland autophagy [29] as well as surplus cell elimination and cell fate specification in the pupal retina [30]–[32]. A remarkable aspect of IRM protein function in this insect is their ability to partially substitute for one another in important morphogenetic events. This has been particularly well demonstrated in the generation of the larval body wall musculature during embryogenesis, where null mutations affecting either *rst* or *kirre* show only very subtle muscle defects, whereas embryos deficient for both genes are also almost completely deficient in myoblast fusion [11]. Partially redundant roles in this process were also recently demonstrated for *hbs* and *sns* [33].

The final steps of Drosophila compound eye morphogenesis constitute an excellent model to investigate the functional interplay between IRM molecules in the context of cell fate specification. In *D. melanogaster* the adult eye is made of about 750 repetitive hexagonal units called ommatidia, each containing eight photoreceptors, four lens-secreting cone cells, three morphologically and functionally distinct groups of pigment cells (pc1, pc2 and pc3), and three mechanosensory bristles. Except for the latter, the specification of these cell types from a previously unpatterned epithelial monolayer - the eye imaginal disc - and their assembly into ommatidial units proceeds sequentially, starting around mid-third larval instar, and can be visualized as a wave of cell differentiation, the morphogenetic furrow, that sweeps the disc from posterior-to-anterior. This process concludes during the first half of pupal development, when some of the still unspecified interommatidial cells (IOCs) are recruited to become pc2 and pc3 cells while the remaining ones undergo apoptosis. The choice between life and death in this specific context seems to rely on the relative position of IOCs, which sort themselves so as to maximize their apical contacts with the border of the already specified pc1 cells [30]. This in turn, occurs concomitantly with, and appears to depend on, the reorganization of Rst protein localization in IOC membranes, which from an initial more or less homogeneous distribution becomes progressively restricted to the IOC/pc1 borders. The inability to correctly promote this Rst protein redistribution, have been shown to block IOC sorting and result in adult eyes containing surplus cells that differentiate into extra pigment cells, thus distorting the ommatidial organization and causing a strong, characteristic “rough” phenotype that gives the gene its name [30], [31], [34] . Nevertheless, some *rst* mutants that appear to almost completely lack Rst protein have compound eyes that are either normal or show only very mild roughness [9], [25] indicating that in certain conditions IOCs may be able to sort themselves out and differentiate normally even in the presence of very low levels of *rst* activity .

Previous work from our group that resulted in the phenotypic and molecular characterization of *rst^D^*, a relatively unstable, semi-dominant regulatory mutation in the *rst* gene, showed that the right timing of Rst redistribution in IOCs appears to be critical not only for the correct elimination of surplus IOCs but also for the process of pc2 and pc3 differentiation, and strongly indicated that precise temporal regulation of *rst* transcription might be one of the key requirements for its correct functioning in the final steps of eye morphogenesis [32]. In this study we further investigated this aspect of *rst* function by analysing its transcriptional dynamics in the developing retina by quantitative real-time PCR, both in wild type and mutant alleles, during IOC sorting and in the stages immediately preceding and following it. Our results support the model previously put forward by Araujo et al [32], who proposed that Rst protein redistribution is consequence of both its selective stability at the border between IOC and pc1s and tightly regulated temporal changes in the *locus* transcriptional rate. Additionally we show that *rst* paralogue *kirre* appears to function redundantly with it in this process, similarly to what has been previously shown for both genes in myoblast fusion during embryogenesis, thus providing an explanation for the weak phenotype often observed in loss-of-expression *rst* alleles. Finally we provide evidence that both genes are co-regulated, having complementary transcriptional dynamics and that RNAi mediated silencing of *rst* leads to a dramatic increase of *kirre* mRNA levels around the time of IOC redistribution. Taken together, these results refine and provide a clearer picture of how the specification of survival and apoptotic fates by differential cell adhesion during the final steps of compound eye morphogenesis in insects are controlled at the transcriptional level.

## Results

### Rst protein redistribution in IOCs correlates with changes in rst mRNA levels

**Figure 1:**
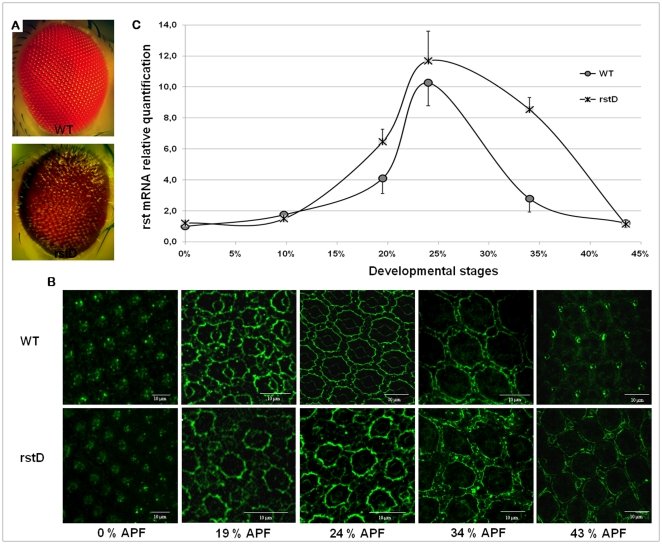
Adult eye phenotypes and protein expression pattern plus mRNA level quantification of *rst* during the first 43% of pupal retina development. (**A**) wild type control (Canton S) and *rst^D^* adult compound eyes. Note the glassy and brownish appearance of the mutant. (**B**) Comparative pattern of Rst protein localization in wild type control and *rst^D^* mutant retinae, visualized by anti–rst Mab 24A.5. In the mutant IOC sorting does not start before 32% APF and the Rst continues to be detected at IOC/pc1 border by 43%APF (**C**) Quantification of relative mRNA levels of *rst* in wild type and *rst^D^* mutant retinae during the first half of pupal development by RT-qPCR. In the mutant, starting around 20% APF,rst mRNA concentration rises sharper and falls significantly slower than in wild type, although both express similar basal levels at the beginning and end of the first half of pupal development. See text for details.

*rst^D^* flies differ from other previously described *rst* mutant alleles by having “glassy”- looking compound eyes with a brownish-red color (Fig. 1A) instead of the usual bright red, rough-like phenotype, by being capable of spontaneously and stably revert both to wild type-like and severe rough eye phenotypes at a moderate frequency (Octacilio-Silva S, Machado M and Ramos RGP, unpublished observations) and by displaying a delay in IOCs intracellular redistribution of Rst protein, which happens 8–10% later in development compared to wild type (Fig. 1B). This delay correlates with late cell sorting, late and erratic apoptotic elimination of surplus IOCs and incomplete pc2 and pc3 differentiation (32). Identification of a genomic rearrangement associated to the *rst^D^* allele and its mapping to the *rst* regulatory region [28], [32] raised the question of how such a mutation could directly affect Rst protein redistribution in IOCs. A relatively simple explanation, based on a combination of differential stability of Rst at the pc1/IOC borders and tightly regulated changes in *rst* transcription around cell sorting stage was proposed [32]. In order to test this model we first compared the relative changes of rst mRNA levels in retinae from both wild type controls and homozygote *rst^D^* mutants isolated between 0 and 44% pupal development using real time quantitative PCR (Fig 1C). In wild type retinae, rst mRNA was initially detected at relatively low levels from 0 to 10% development after puparium formation (AFP). By 19% AFP however, a 4-fold increase in these basal levels was observed, reaching over 10-fold at about 24% APF. From this peak until about 34% AFP rst mRNA levels steadily decreased by about 3-fold before reaching basal levels again at 43% APF. The temporal dynamics of rst mRNA concentration in retinas from *rst^D^* pupae, on the other hand, followed a similar pattern until 24% AFP, although the initial rise tended to be steeper and peak levels were consistently higher than in wild-type. From then on, however, rst mRNA concentration remained high until at least 34% APF, when it slowly started falling to eventually reach about the same basal levels as wild type (Fig. 1C). These results correlate directly with the temporal course of Rst protein redistribution in IOCs shown in figure 1B, both in mutant and wild type, further corroborating the idea that this phenomenon is directly regulated at the transcriptional level while providing a simple explanation for the delay in IOC sorting observed in rst^D^ mutants.

### The absence of a wild type-like temporal dynamics of rst mRNA concentration does not necessarily prevent the normal organization of the adult compound eye

**Figure 2:**
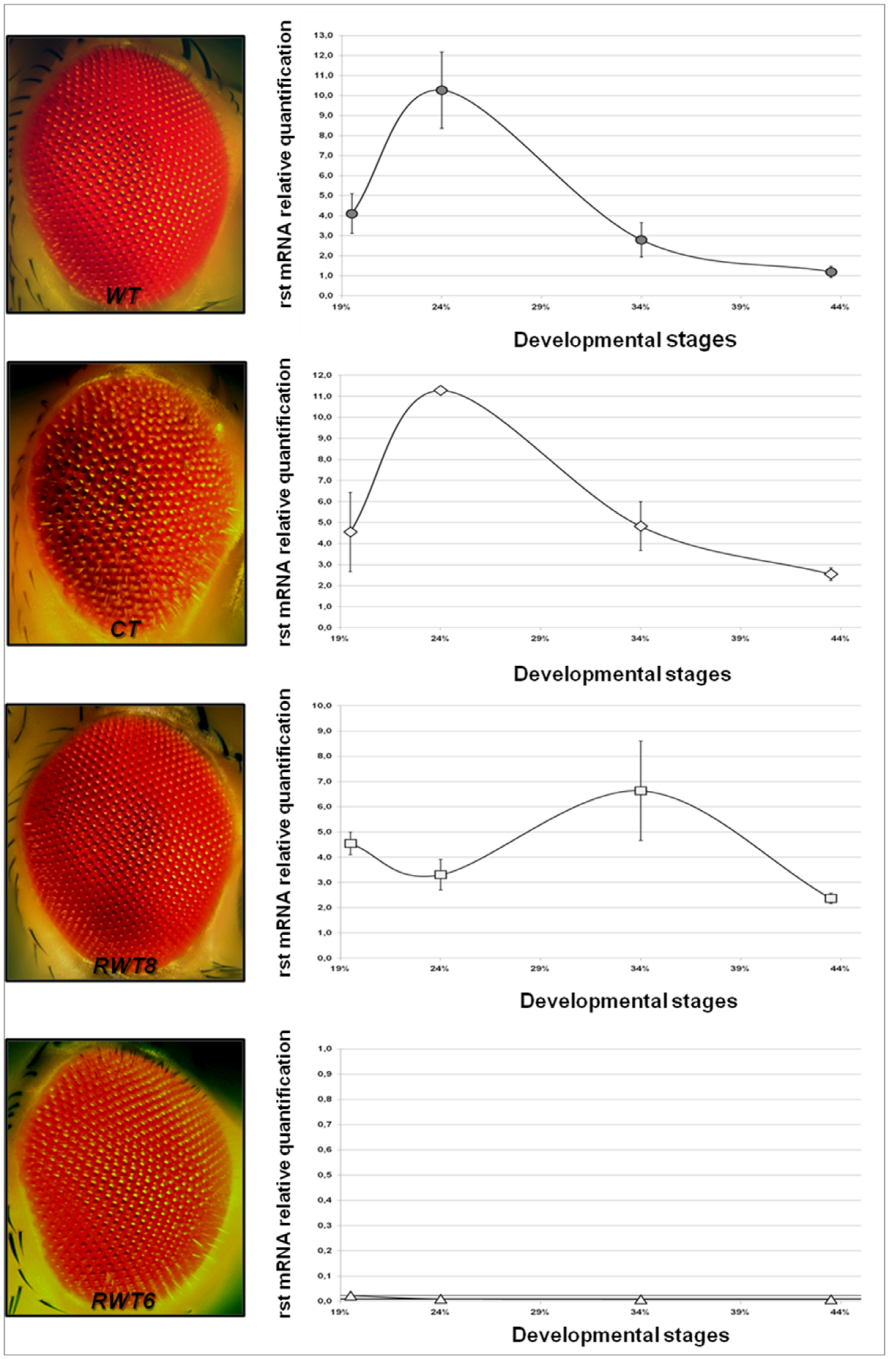
RT-qPCR quantification of relative rst mRNA levels in *rst^CT^* mutant and two *rst^D^* wild type-like revertants together with corresponding eye phenotypes. Despite its severe rough eye phenotype, *rst^CT^* (which expresses a truncated version of the Rst protein) shows an essentially wild type rst mRNA temporal profile. Both *rst^D^* wild type-like revertants however, are defective in rst mRNA expression, which is completely absent in *RTW6* and displays a striking departure of wild type temporal profile in *RTW8*, showing that the requirement for *rst* wild type function can be bypassed in these mutants during pupal eye development.

If, as the previous experiment imply, *rst* transcriptional activity directly impacts on the correct timing of Rst protein redistribution in IOCs, therefore promoting correct IOC sorting, then *rst^D^* mutants reverting to wild type-like eye phenotypes should also revert to a wild type-like temporal profile of rst mRNA expression. To this end we investigated the retinal rst mRNA concentration dynamics of two independently isolated *rst^D^* wild type-like revertants, *RWT6* and *RWT8*, in the same conditions and time windows previously described for *rst^D^*. Additionally we performed the same analyses in *rst^CT^* mutants, which carry a small deletion in the coding region of an otherwise normal mRNA, causing the production of a Rst protein truncated at its intracellular domain. This correlates phenotypically with a block in cell sorting, lack of elimination of surplus IOCs and a severe rough eye phenotype [9], [31] (See also Fig. 2, left). The truncated Rst^CT^ polypeptide shows a timing of expression indistinguishable from wild type but fails to accumulate at the pc1/IOCs borders, collecting instead in intracellular endocytic vesicles [31], therefore allowing us to test whether the destabilization of the Rst protein from the membrane could in any way modulate rst mRNA levels during cell sorting stage. As expected the temporal profile of Rst mRNA concentration in *rst^CT^* mutants closely follows the wild type pattern, although the decrease after the 24% APF peak appears to be somewhat slower in the mutant (Fig. 2).

The analyses of the two *rst^D^* revertants, on the other hand, gave surprising results. Despite both having essentially normal adult compound eyes, their mRNA concentration dynamics differed strikingly from wild type during IOC sorting stage and in the developmental time interval immediately following it (Fig. 2). *RWT8*, for instance, showed actually a decrease in rst mRNA concentration at 24% APF, compared to 19%, and peaked at about 34% APF, reaching levels above wild type at this same time point, while in *RWT6* we were unable to detect retinal rst mRNA expression at any time point from 0 to 44% APF. This latter finding, implying complete lack of *rst* function in the eye, does not appear to be an artifact due, for instance, to the absence of specific sequences recognized by the primers used for the RT-qPCR experiments, since rst mRNA was detected in *RWT6* embryos and in post embryonic tissues such as salivary glands submitted to the same experimental conditions. Besides, no immunoreactivity can be seen in *RWT6* pupal retinae treated with anti-rst monoclonal antibody Mab 25A.4 (data not shown). Taken together these data lead to the intriguing implication that, at least in some circumstances, *rst* expression might be dispensable for correct ommatidial patterning.

### *kirre* can substitute for *rst* function during compound eye development

**Figure 3:**
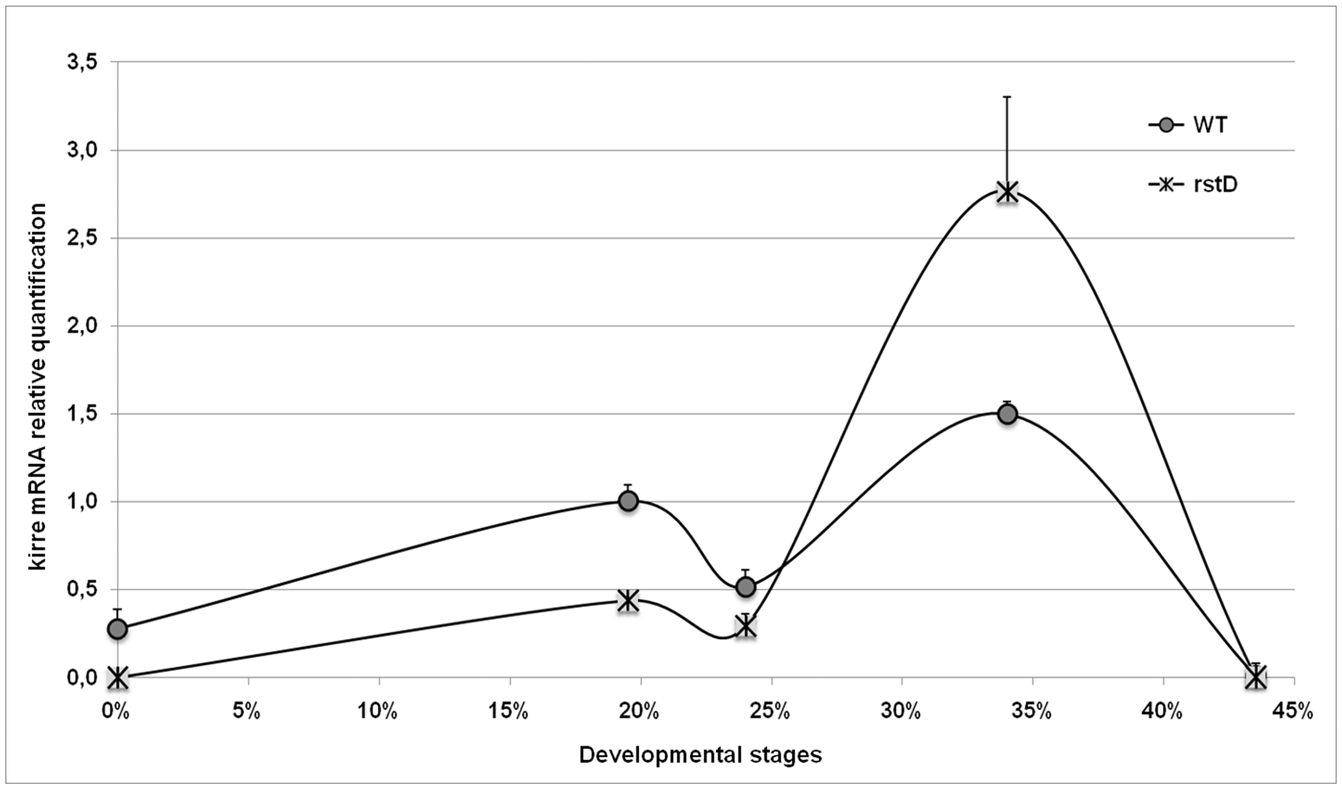
Comparative temporal quantification of relative kirre mRNA levels in wild type and *rst^D^.* The symbols representing each genotype are indicated in the graph. Although qualitatively similar to wild type, kirre mRNA levels in *rst^D^* retinae reach significantly higher concentrations at their 34% APF expression peak when compared to their basal levels.

**Figure 4:**
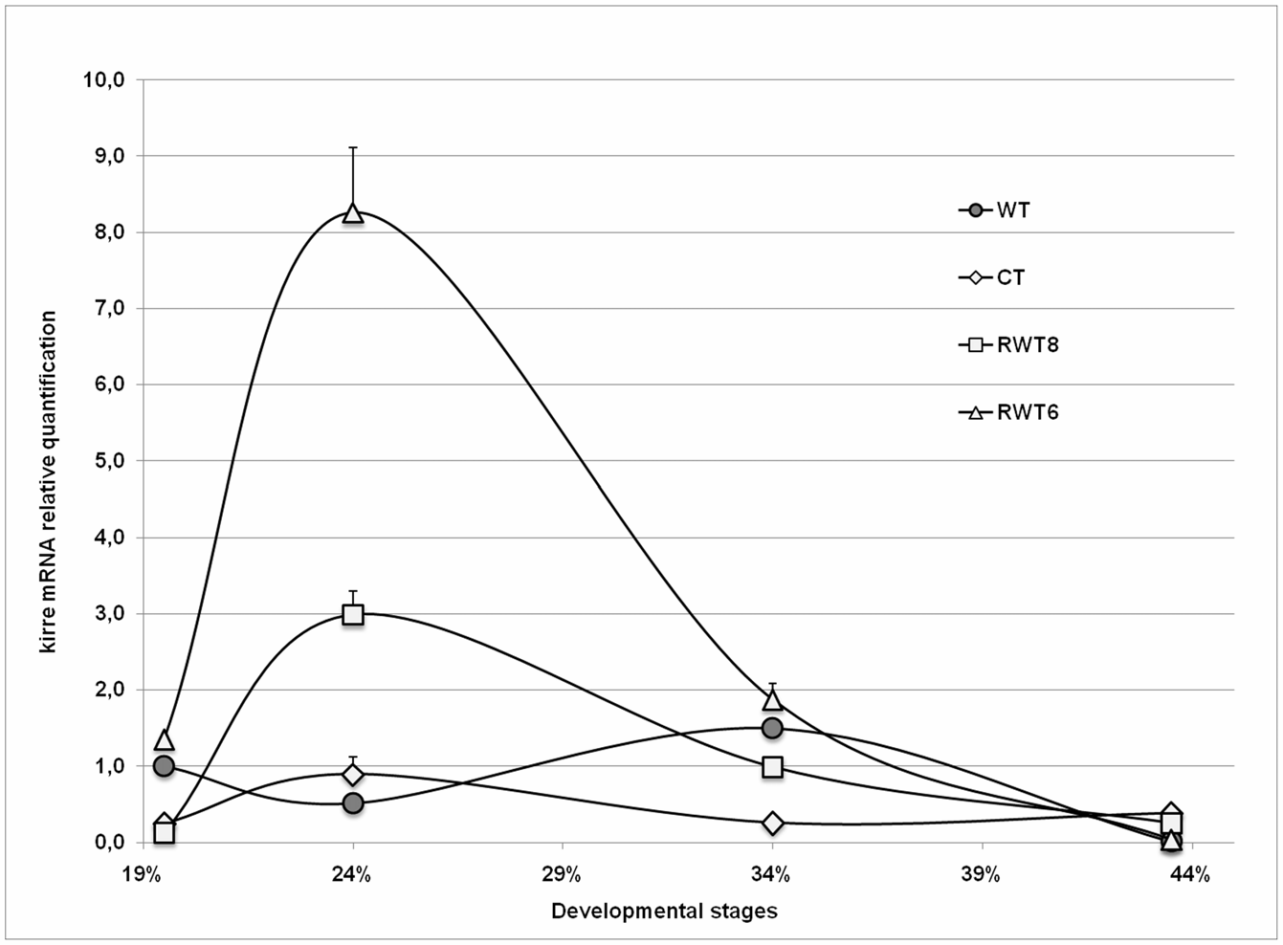
Comparative temporal quantification of relative kirre mRNA levels in wild type, *rst^CT^* and the two *rst^D^* wild type-like revertants. The symbols representing each genotype are indicated. All mutants show a “rst-like” temporal profile of kirre mRNA expression, although in the case of rst^CT^, changes in concentration relative to wild type are very small.

The apparent paradox posed by *rst^D^* revertants having normal compound eyes despite lacking *rst* expression in the pupal retina could be easily resolved by assuming that at least some *rst* functions during eye development could be equally performed by its paralogue *kirre*, similarly to what has been demonstrated for myoblast fusion in the embryo [11]. To investigate this possibility we repeated the RT-qPCR analyses presented in Figures 1 and 2, this time using *kirre* specific primers. A comparison between kirre mRNA temporal profiles of wild type and *rst^D^* is shown in Figure 3. In wild type retinae, absolute levels of kirre mRNA were much lower than *rst* and remained relatively constant throughout the time period analysed. Nevertheless significant departures from the basal level did occur, with the lowest levels coinciding with the peak of rst mRNA expression, at about 24% APF and highest around 34% APF when rst mRNA concentration approached again basal levels. The same qualitative profile was displayed by *rst^D^* retinae although here also, peak levels were higher than in wild type. In contrast, quantification of kirre mRNA in the retinae of the two *rst^D^* revertants (Fig. 4) showed a completely different pattern, much closer to that shown for rst mRNA in wild type, with a sharp increase around the time of cell sorting followed by a return to near basal levels by 34% AFP. This is particularly clear in *RTW6* retinae where the temporal dynamics of kirre mRNA shown in Figure 4 is almost indistinguishable from that of wild-type rst mRNA (Fig. 2). It is interesting to note that even in *rst^CT^* retinae, despite its wild type-like levels and low quantitative variations, kirre mRNA concentration tended to follow a *rst*-like temporal distribution, peaking at 24% instead of 34% AFP (Fig. 4).

**Figure 5:**
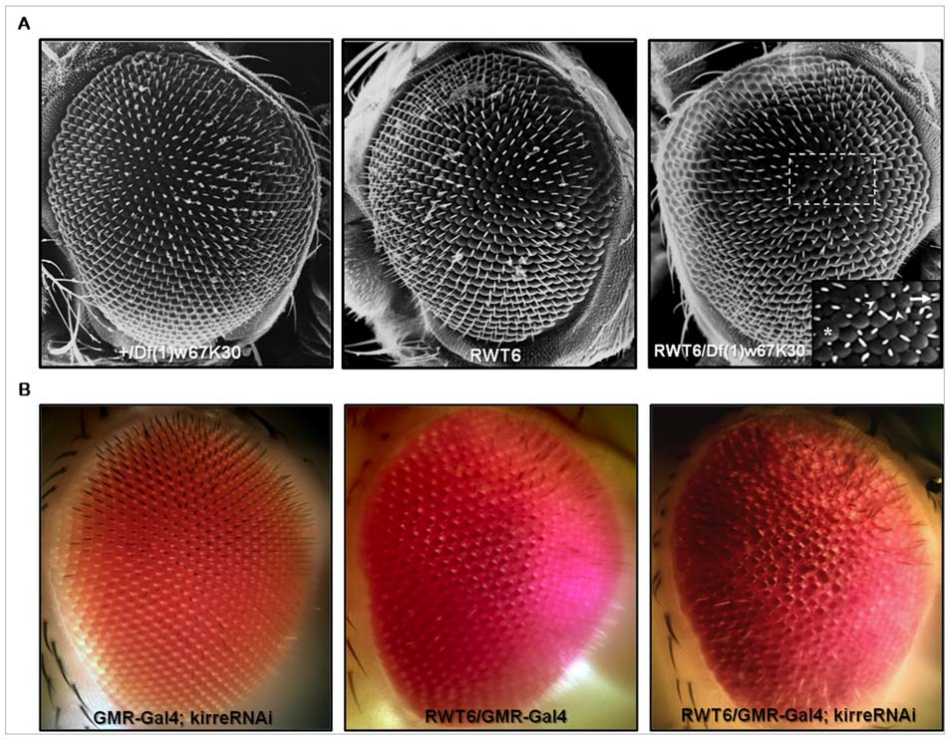
Genetically lowering *kirre* dosage in the *RTW6* revertant causes defects in adult ommatidial organization. (**A**) Scanning electron micrographs. Compound eye phenotype from a *RTW6/Df(1)w^67k30^* heterozygote female, compared to those from her parent genotypes, *RTW6/Y* and *Df((1)w^67k30^)/+*. Leaving only the *kirre* copy present in the RTW6 chromosome when no functional copies of *rst* are present in the eye lead to a mild but clear disruption of ommatidial patterning showed in the inset: lack or mispositioning of mechanosensory bristles (arrowheads), bristle duplications (arrow) and fused ommatidia (asterisk). (**B**) Dissecting stereomicroscope images. Head from a *RTW6* male where *kirre* function has been almost completely knocked down by expressing one copy of the *UAS-kirre RNAi 3111* transgene under the control of the *GMR-Gal4* eye driver (see methods). Eyes from a male carrying only the driver and a *RWT6*/Y are also shown. Note the clear increase in the severity of the phenotype, particularly the higher number of fused ommatidia and mispositioned bristles, compared to the *RTW6/Df(1)w^67k30^*eye in (**A**).

The drastic quantitative and qualitative changes in kirre mRNA levels seen in *rst^D^* revertants, particularly in *RTW6*, when compared to wild type controls, although compelling, do not directly demonstrate the ability of *kirre* to rescue *rst* function during IOC sorting. To this end we genetically lowered *kirre* dosage in *RWT6* mutants by crossing them to flies carrying the *Df(1)w^67k30^* deletion which removes both *rst* and *kirre* genes [11]. Figure 5A shows scanning electron micrographs of compound eyes from the parental strains and the relevant progeny. Homozygote individuals for this deficiency die during embryogenesis with severe defects in muscle development but heterozygotes, being *rst^+^ kirre^+^*/*rst^−^ kirre^−^* are viable and have normal compound eyes, as do the *rst^−^ kirre^+^*/*rst^−^ kirre^+^* homozygote *RTW6* flies. However, trans-heterozygotes *Df(1)w^67k30^*/*RTW6*, genotipically *rst^−^ kirre^−^*/*rst^−^ kirre^+^* display mild but characteristic ommatidial defects present in other *rst* mutants, including irregularly spaced bristles and fused ommatidia (inset). The relatively weak phenotype observed could be a consequence of the high levels of kirre mRNA produced in the retina by the remaining genomic copy of *kirre*, but could also be an indication that the eye phenotype rescue seen in *RTW6* revertants was only in part due to *kirre* redundant function. To test this possibility we used an *UAS-kirre RNAi* construct driven by *GMR Gal4* to knock down *kirre* function in *RTW6* retinae. As shown in Figure 5B, a clear increase in the severity of the phenotype is observed, particularly in the irregular spacing and the number of ommatidial fusions. Taken together the results show that high levels of *kirre* expression alone at the time of IOC sorting can bypass the requirement for *rst* function in this process and provide strong support for the idea that both genes could act redundantly also during eye development.

### Post transcriptional silencing of *rst* in the pupal retina leads to an upregulation of kirre mRNA during cell sorting stage

**Figure 6:**
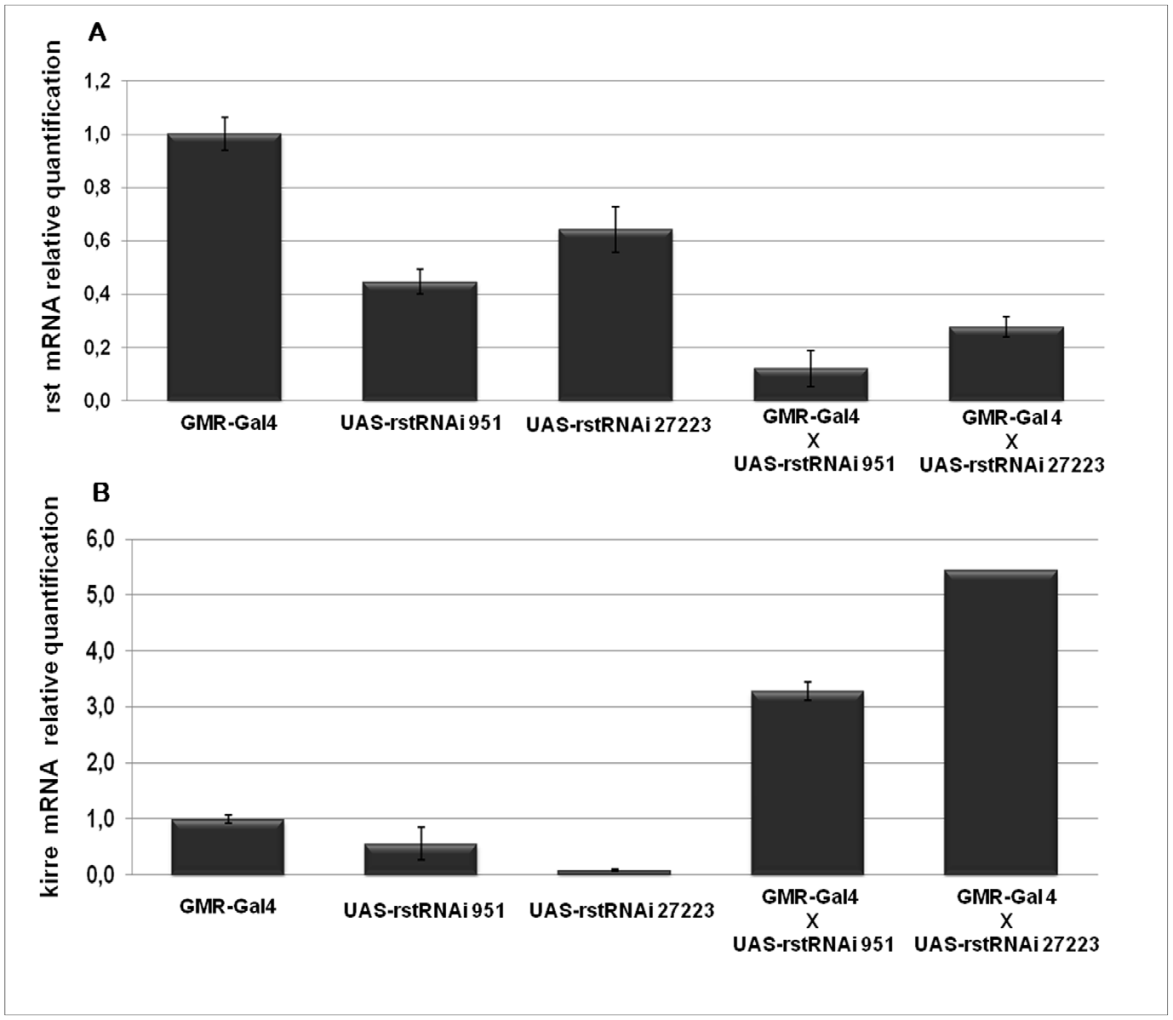
*rst* RNAi silencing leads to an increase of kirre mRNA levels at the time of cell sorting. (**A**) Relative quantification of rst mRNA levels at 24% APF in the retinae expressing one copy of either *UAS-rstRNAi 951* or *UAS-rstRNAi 27223* transgenes, driven by *GMR-Gal4*, compared to those expressing either the driver alone or the transgenes alone. (**B**) Relative quantification of kirre mRNA levels at 24% APF in the retina of the flies with the same genotypes as in (**A**). A clear increase in kirre mRNA levels can be seen in the *rst*-silenced retinae.

The quantitative and qualitative differences in kirre mRNA concentration profile compared to wild type, found in *rst^D^* revertants in the absence of normal rst mRNA levels during the 19–34% APF time window, together with their almost perfectly complementary timing of expression, leads naturally to the suggestion that transcription of both genes might be co-regulated and, somehow sensitive to each other's RNA or protein levels. Moreover *rst* and *kirre* both map next to each other in the genome and are transcribed in opposite directions [11], making it possible for them, at least in principle, to share common regulatory elements. However, since the highly coordinated compensatory changes in the mRNA levels of both genes shown in the previous experiments could be clearly demonstrated only in mutants that previously had gone through reversion events involving genomic rearrangements in the *rst* regulatory region, it was conceivable that they could reflect a “lucky accident” caused by a favorable reorganization of regulatory sequences in the DNA of *RTW6* and *RTW8* rather than a true physiological response. We therefore asked whether by deliberately lowering rst mRNA peak levels in wild type retinae we could bring about a corresponding increase in kirre mRNA. To this end we quantified the levels of rst and kirre mRNA at 24% APF in wild-type retinas that expressed either one of two independently generated *rst RNAi* constructs under the control of a *GMR-Gal4* driver (Fig. 6). Activation of these constructs in wild type retinae lead to reduction of rst mRNA concentration to levels between 20 and 35% of average parental levels quantified in the same conditions (Fig. 6A) although only very mild defects were apparent in adult individuals (data not shown). Quantification of kirre mRNA however showed that such reduction in rst mRNA levels correlated with a 5 to 10 fold increase in the relative concentration of kirre mRNA compared to average parental levels (Fig. 6B), thus providing strong evidence that both genes are indeed coordinately regulated.

**Figure 7:**
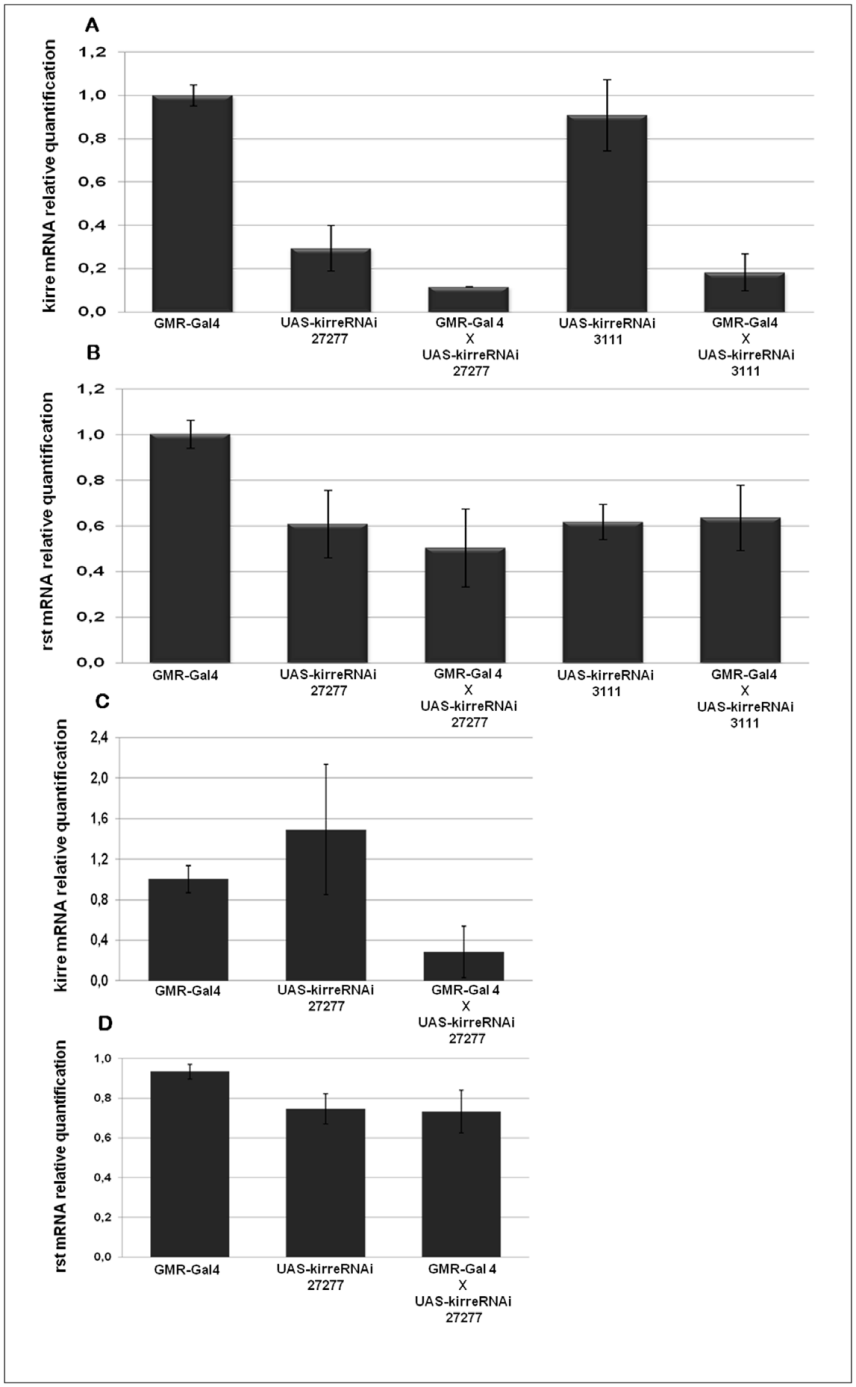
*kirre* silencing does not appear to influence rst mRNA concentration in the retina. (**A**) Relative quantification of kirre mRNA levels at 24% APF in retinae expressing one copy of either *UAS-kirreRNAi 3111* or *UAS-kirreRNAi 27277* transgenes, driven by *GMR-Gal4*, compared to those expressing the driver alone or the transgenes alone. (**B**) Relative quantification of rst mRNA levels at 24% APF in the retinae of the flies with the same genotypes as in (**A**). (**C**) Relative quantification of kirre mRNA levels at 34% APF in retinae expressing one copy of *UAS-kirreRNAi 27277*, driven by *GMR-Gal4*, compared to those expressing the driver alone or the transgene alone. (**D**) Relative quantification of rst mRNA levels at 34% APF in retinae with the same genotypes as in (**C**). In neither time point significant differences in rst mRNA levels between *kirre*-silenced retinas and the controls could be detected.

Once we were able to show that reduction in rst mRNA concentration can induce a compensatory increase of mRNA kirre levels, we asked if the converse was also true and compensatory changes in rst mRNA levels could be elicited in *kirre* silenced retinae. The results presented in Figure 7 do not support this assumption. Despite a reduction of retinal kirre mRNA levels to less than 25% of average parental levels we did not detect significant changes in rst mRNA, either at its 24% APF peak or at 34% APF, that coincides with kirre mRNA highest levels, indicating that lowering *kirre* expression has no effect on rst mRNA concentration in this tissue.

## Discussion

The ability of interommatidial cells to reorganize their apical contacts such as to maximize their membrane interactions with primary pigment cells, around 24% AFP, is a key step for generating the highly precise geometrical pattern of the adult compound eye, but it is also needed to create differences in intercellular adhesion and signaling that allow the final cell fate specification decision in the pupal retina - that between survival and programmed cell death – to correctly take place. A central involvement of Rst cell adhesion molecule in the IOC reorganization event has been inferred not only from the observation that this latter process paralleled Rst protein redistribution in IOC membranes but also from two independent sets of evidence: first, that the complete absence or truncation of *rst* protein product often blocked IOC sorting and lead to an “all to one” switch in cell fate, with surplus pigment cells appearing in the adult eye as consequence of lack of cell death [30], [31]; second, that when Rst redistribution was delayed, so was IOC sorting, what produced an “all to none” response in which the survival versus death choice is either not correctly made or not properly implemented, leading to surplus cells dying later and erratically while the remaining ones failed to differentiate into proper pigment cells [32]. These data emphasized the need for *rst* function not only at the right place but also at the right time, and implied very precise spatial and temporal controls of its expression. Unraveling the details of these control mechanisms is therefore an essential prerequisite for a fuller understanding of the nature and dynamics of the signals to which cells must respond to die or to differentiate in the final steps of ommatidial patterning. The quantitative analysis presented here showed a striking correlation between rst mRNA concentration over time and the qualitative dynamics of Rst protein localization in IOC membrane, both in wild type and in the regulatory *rst^D^* mutant thus implicating the temporal control of *rst* transcription, rather than a reshuffling of Rst protein molecules previously present in the membrane, as a main factor responsible for the critical changes in cell adhesion specificity that allow IOC sorting to take place. These findings help to shed light on a little studied and, so far, underestimated aspect of eye development, adding a new dimension to the complex process of ommatidial patterning and differentiation. Besides, our results demonstrate functionally the ability of *kirre* to rescue *rst* function in IOC sorting. Although the possibility of redundant roles for *rst* and *kirre* in retinal development had been previously suggested, supporting evidence was mainly indirect and obtained from either protein co-localization or overexpression experiments (reviewed in [8]). More recently, Bao et al [35] provided convincing evidence, based on the analysis of retinal patterning at the time of IOC sorting, that Rst and Kirre function redundantly to maintain the spacing between developing ommatidial groups. We have extended these observations by directly showing the ability of high levels of *kirre* expression in mutants with no or very little rst mRNA to bypass the need for *rst* function, including its influence in cell fate choice. The molecular mechanism underlying this redundancy, however, remains to be elucidated. Rst and Kirre have highly conserved extracellular domains [11], co-localize in IOCs and both appear to bind Hbs at the border with pc1s [8], [35], – although in the case of Kirre direct evidence for this latter assertion is missing – which seems to imply that the two proteins are fully interchangeable in their interactions with other extracellular and intracellular molecules during retinal development. However their intracellular domains show very little similarity [11] suggesting that they might have few, if any, common cytoplasmic binding partners. Genetic and biochemical studies aiming at identifying possible intracellular interactors of Kirre and Rst have been performed in different tissues, uncovering sets of directly binding proteins and potential signaling pathways that are distinct for each gene product [36]–[38]. Since the intracellular domain of Rst is required for its function in the pupal retina [31], probably by interacting with actin cytoskeleton [29], [39], it would be important to ascertain whether *rst*/*kirre* functional redundancy in this context results from their ability to act through the same intracellular pathways or it is a consequence of their interaction with different ensembles of intracellular targets that can nevertheless lead to the same end result.

Perhaps the most interesting finding presented here is the evidence for compensatory co-regulation of rst and kirre mRNA concentrations and its asymmetrical nature. A possible, although unlikely explanation for this asymmetry could reside in the much higher absolute levels of rst mRNA present during the temporal period examined, making small changes in relative concentration easier to detect with confidence. Also we have neither investigated the effect of overexpression of one gene on the concentration of the other, nor whether similar coordination in mRNA expression is taking places in other developmental contexts where complementary or redundant functioning of *rst* and *kirre* seem to be required, such as myoblast fusion [11], salivary gland [29] and optic lobe development [40]. In this latter context it is worth mentioning that *RTW6*, but neither *RTW8* nor *rst^D^*, show axonal pathfinding defects in the optic lobe (Machado M, Octacílio-Silva S and Ramos, R unpublished observations) suggesting that at least in some developmental contexts *rst* and *kirre* might not be fully redundant.

Finally, an intriguing aspect of the compensatory response reported here is that it implies some kind of sensor mechanism capable to post-transcriptionally read *rst* expression levels and adjust those of *kirre* accordingly. Here again mRNA, rather than protein concentration, may play the main role, since in *rst^CT^* low levels of *rst* activity caused by the truncated intracellular domain and destabilization of the protein from the membrane leads only to a minimal, if any, increase of kirre mRNA levels at 24% APF. Also it is conceivable that rather than upregulating kirre transcription, a decrease in degradation could be taking place, leading to an accumulation of kirre mRNA molecules. Experiments designed to further test this possibility as well as to map rst mRNA sequences that might be relevant for this putative regulatory feedback loop between *rst* and *kirre* are currently underway. Whatever the case, these findings suggest a general mechanism by which the expression both genes could be fine tuned to optimize their redundant roles during development.

## Materials and Methods

### Fly stocks and genetic crosses

Fly manipulations were performed in vials containing standard Drosophila food at 25°C in a temperature-controlled room. RNAi transgenic stocks *UAS-rstRNAi27223*, *UAS-rstRNAi951*, *UAS-kirreRNAi3111* and *UAS-kirreRNAi27277* were obtained from the Vienna Drosophila RNAi stock center [41]. *rst^D^* revertant stocks *RTW6*/*FM7i* and *RTW8* were isolated in our laboratory as spontaneous reversions to wild-type eye phenotype in a homozygous *rst^D^* stock on a Canton S background that additionally were *rst^D^* dominant suppressors. Genomic organization of RTW6 in the region where the *rst^D^* rearrangement was mapped [32] is indistinguishable from wild type, as assessed by Southern Blot analysis, while *RTW8* appears to have resulted from a 1 kb internal deletion in the rearranged area. Both mutants are homozygous viable but *RWT6/RWT6* females are sterile. All other wild type and mutant stocks used in this study were previously described [9], [11], [32]. *Df(1)67k30*/*RTW6* flies were generated by selecting the non-*Bar* female progeny from a cross between *RTW*6/Y males and *Df(1)67k30/FM7* females. Flies specifically expressing rst or kirre RNAi in pupal retinae resulted from the progeny of crosses between *UAS-RNAi* stocks described above and *GMR-Gal 4* flies [42]. Flies genotypically *RTW6/Y; GMR-Gal4/UAS-kirre-RNA3111* were obtained by first constructing *RTW6/FM7i; CyO/UAS-kirre-RNA3111* females, crossing them to *GMR-Gal4* homozygote males carrying the transgene in the second chromosome and selecting for non-Bar, non Curly males. Flies were examined under a Leica MZFLIII dissecting stereomicroscope equipped with Leica DFC 500 digital camera and acquired images were processed using a Leica Application Suite V3.1.0 software**.**

### Histological and Immunohistochemical methods

lmmunohistochemistry of staged pupal retinae with monoclonal antibody 24A5 [26] was performed as described previously [32], [43] and analysed in a Leica SP5 Confocal microscope. Fly heads were prepared for scanning electron microscopy by ethanol substitution followed by critical point drying and gold coating. Image acquisition was done in a Jeol JSM5200 scanning electron microscope.

### Quantitative Real Time Polimerase Chain Reaction

The two retinae from a single staged pupa were dissected in PBS and while one retina was processed for immunohistochemistry with anti-Rst as a control, the other was immediately transferred into Trizol reagent (Invitrogen) for RNA extraction according to the manufacturer's specification. Genomic DNA was removed by treatment with DNase I (Promega). Concentration and purity of total RNA samples were determined with NanoDrop spectrophotometer (Thermo Scientific, USA). Only Samples with optical density absorption ratio A260/A280 between 1.8 and 2.2 were taken for cDNA synthesis. The High-Capacity Reverse Transcription Kit (Applied Biosystems) was used for the reverse-transcription reaction with the concentration of 200–400 ng RNA sample in accordance with the manufacturer's protocol. RT-qPCR reactions were performed mostly in three biological and technical replicates in a 96-well plate (Applied Biosystems) using the Gene Amp® 7500 Sequence Detection System (PE Applied Biosystems) with Sybr Green Rox Plus (LGC Biotechnology) and Sybr Green PCR Master Mix (Applied Biosystems), for the experiments involving RNAi silencing. The primers sequences were: QPCR-RST-Forward CG4125∶3 Type exon 5′– TGCCACCGAGGATCGCAAAG; QPCR-RST-Reverse CG4125∶4 Type exon 5′– GCAGCGGTATCACCGTGTAC; QPCR-KIRRE-Forward CG3653∶6 5′- CGGTGCACAAAATGCAGTTAC and QPCR-KIRRE-Reverse CG3653∶6/7 5′- GCTAACTCAGGAAGCGAAAT. All reactions were run at 95°C for 10 minutes, then 40–45 cycles of 95°C for 15 seconds and 60°C for 1 minute. RpL32A was used as reference and amplified using primers QPCR-RPL32A-Forward CG7939 5′– TCGACAACAGAGTGCGTCGC and QPCR-RPL32A-Reverse CG7939 5′– CTTGAATCCGGTGGGCAGCA. To check amplification efficiency, calibration curves for both target and reference genes were performed. Reactions without template were run in parallel for all plates as internal controls. A melting curve analysis was made after each run to confirm the specificity of the amplification. The ΔΔC_q_ method [44] was used to compare the *rst* expression between wild type and mutants. *rst* transcript levels were normalized with *RpL32A* transcript levels in the same cDNA templates and used as indicators of the expression levels. Data points represent the average of independent biological samples (three in general) with error bars corresponding to standard deviations.
